# Postoperative complications from alcohol use disorder in an adult congenital heart disease patient: a case report emphasizing the role of high-complexity congenital centers

**DOI:** 10.1186/s13019-026-04595-8

**Published:** 2026-08-14

**Authors:** Benjamin Alexander, Lawrence Greiten, Emily Sanders

**Affiliations:** 1 Department of Pediatric Cardiology and Adult Congenital Heart Disease, Arkansas Children’s Hospital, 1 Children’s Way, Little Rock, 72202 USA; 2 Cardiology, University of Arkansas for Medical Sciences, 4301 West Markham Street, Arkansas 72205 Little Rock, USA

## Abstract

**Background:**

Superior sinus venous atrial septal defect (SVASD) associated with partial anomalous pulmonary venous return (PAPVR) is a rare congenital heart disease that is predominantly diagnosed and surgically treated in childhood; however, sometimes the diagnosis is delayed and requires intervention in adulthood.

**Case presentation:**

A 69-year-old patient presented to the adult congenital heart disease (ACHD) clinic due to shortness of breath and recurrent syncopal episodes and was found to have significant right ventricular enlargement by echocardiogram of undetermined cause. Cardiac CT detected a large SVASD with PAPVR that required surgical intervention. The postoperative course was complicated by delirium tremens.

**Conclusions:**

ACHD patients often have many comorbidities that are not commonly seen in the pediatric population. Because of this, it is of utmost importance that pediatric surgical centers offering procedures for ACHD patients have established protocols to manage these comorbidities postoperatively, including substance withdrawal symptoms.

**Supplementary Information:**

The online version contains supplementary material available at https://doi.org/10.1186/s13019-026-04595-8.

## Introduction

A superior sinus venosus atrial septal defect (SVASD) is a rare congenital condition where the superior vena cava (SVC) overrides the atrial septum due to deficiency of the common wall between the SVC and the right-sided pulmonary veins; SVASD is commonly associated with partial anomalous pulmonary venous return (PAPVR) of one to three pulmonary veins (PVs), most commonly involving the right upper pulmonary vein returning directly to the SVC [1]. In general, SVASD and PAPVR result in a large left-to-right shunt, and, if not corrected by surgical or transcatheter intervention, leads to pulmonary overcirculation, right ventricular (RV) dilation and remodeling, and eventual development of pulmonary arterial hypertension. With these known long-term complications, SVASD and PAPVR is usually diagnosed and repaired during childhood; however, as symptoms do not typically develop until adulthood, late diagnosis and repair are not uncommon. These surgeries are typically done at pediatric hospitals by pediatric heart surgeons [2]. Many adult with congenital heart disease (ACHD) patients have comorbidities not commonly encountered in the pediatric population that will require subspecialty care or established protocols for management of those comorbidities during their inpatient, postoperative course.

Here, we present a case of a 69-year-old patient with SVASD and PAPVR of the right upper and two middle PVs to the SVC requiring surgical repair whose postoperative course was complicated by delirium tremens; we also discuss the development of protocol-driven management of alcohol use disorder.

## Case report

**Fig. 1:**
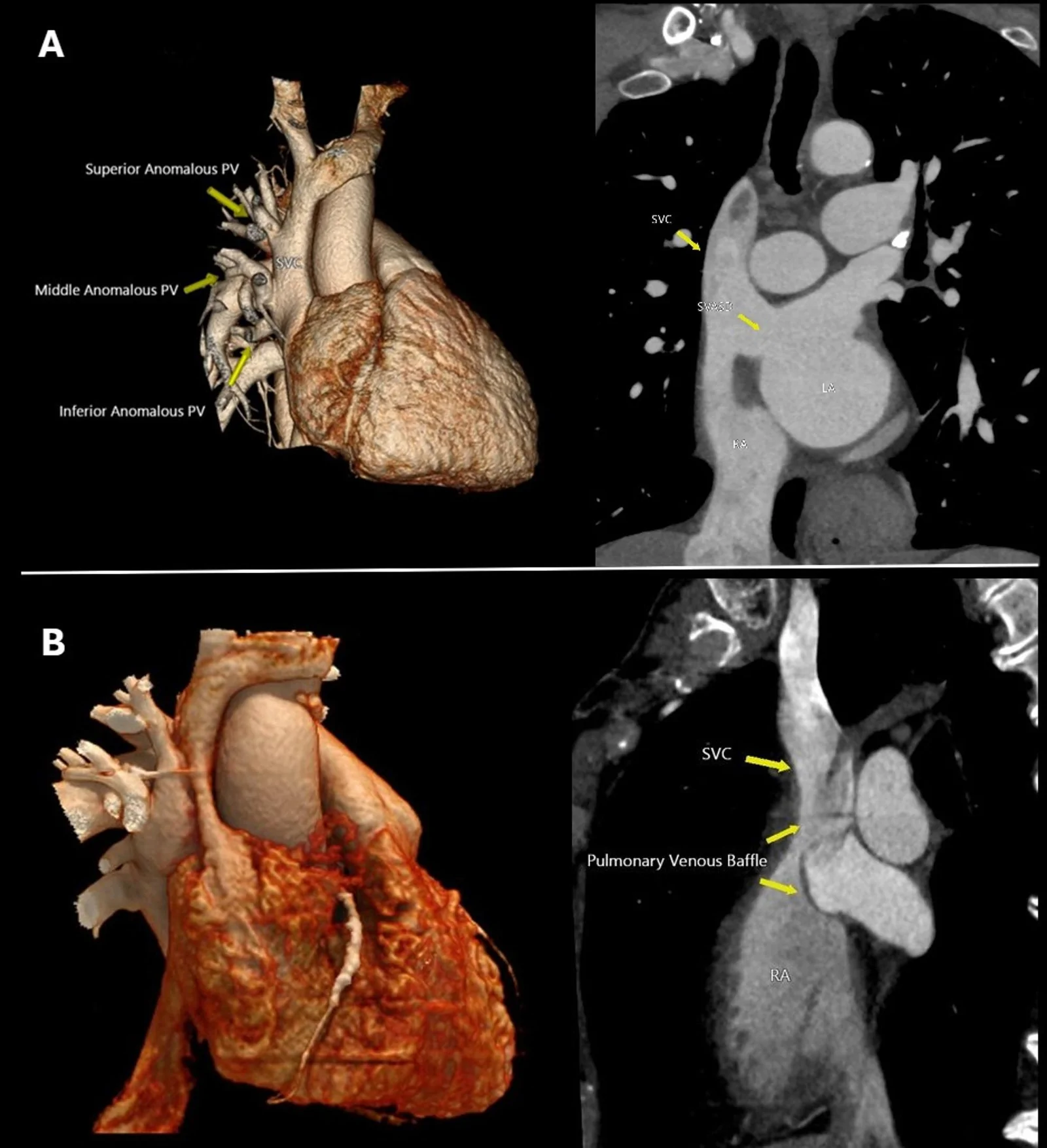
Panel **A** depicts the preoperative 3D and 2D cardiac CT reconstructions highlighting the right-sided anomalous pulmonary veins and the large superior sinus venosus defect. Panel **B** shows the postoperative 3D and 2D cardiac CT reconstructions after baffling the anomalous pulmonary veins to the left atrium and closure of the sinus venosus defect

A 69-year-old patient presented to the ACHD clinic with progressive shortness of breath and recurrent syncopal episodes not associated with palpitations. The echocardiogram showed significant right heart enlargement of undetermined cause with normal right ventricular systolic pressure and preserved systolic function, so advanced imaging was recommended. Cardiac CT was obtained and demonstrated a large SVASD (2.1 cm x 1.7 cm) and anomalous drainage of three right-sided PVs to the SVC or SVC/RA junction. (Fig. 1) Given the patient’s significant symptoms, surgical repair was recommended. The patient had no other known comorbidities; however, on their preoperative outpatient visit, they did report drinking 2–3 beers per night for approximately the last 20 years. They denied any history of tremors, diaphoresis, or seizures when abstaining from alcohol. Preoperative labs including liver enzymes, platelets, and INR were normal.

The patient underwent baffling of the anomalous PVs through the sinus venosus ASD to the left atrium and closure of remaining ASD with bypass time of 71 min and cross clamp time of 53 min. Postoperative transesophageal echocardiogram revealed no residual ASD and unobstructed pulmonary venous baffle.

The postoperative course was complicated by significant agitation, nausea, and headache shortly after arrival to the postoperative unit. Around 4 h postop, the patient experienced a tonic-clonic seizure. The patient underwent emergent head CT to rule out acute intracranial hemorrhage, which was negative for acute bleed. The patient had a total of three seizures over the next hour which responded well to intravenous (IV) midazolam. After discussion with the family, the patient had not had any alcohol in approximately 48 h, which raised concern for delirium tremens. The patient was started on IV thiamine, IV folate, and an oral gabapentin taper at 800 mg three times a day. Within a few hours of initiating treatment for alcohol withdrawal syndrome, the patient had resolution of their seizures and returned to their neurologic baseline. The rest of the postoperative course was uneventful, and the patient was able to be discharged on postoperative day six.

## Conclusions

Surgical repair of SVASD and PAPVR in ACHD patients is often performed at pediatric high-complexity congenital centers by pediatric heart surgeons. ACHD patients often have multiple comorbidities not commonly seen in the pediatric population, including high rates of substance use disorders, found to be almost 3% of postoperative ACHD patients [2–3]. With this in mind, it is extremely important for pediatric hospitals that regularly care for ACHD patients to have preoperative screening specifically for current substance use/abuse. If preoperative screening detects alcohol use disorder, pediatric hospitals should have inpatient protocols in place for identifying and managing alcohol withdrawal syndrome, most commonly with the Clinical Institute Withdrawal Assessment for Alcohol (CIWA) scoring system [4]. In this case, our patient screened positive for alcohol misuse preoperatively (with low-risk consumption per AUDIT-C) [5] without history of alcohol withdrawal symptoms with abrupt cessation as an outpatient. With the known alcohol misuse, the patient was quickly placed on IV thiamine, IV folate, and a gabapentin taper after initial control of seizure activity with IV benzodiazepines. After this patient experience, our institution developed an inpatient protocol for alcohol withdrawal assessment and tiers of withdrawal treatment based on CIWA scores (Supplemental Figure).

While it could be argued that these ACHD patients with multiple comorbidities could be managed in adult hospitals, a study looking at postoperative ACHD outcomes demonstrated lower in-hospital mortality when the patients had their procedures performed by pediatric heart surgeons compared to non-pediatric heart surgeons [6].

With modern surgical techniques, there are now more adults than children living with CHD. SVASD and PAPVR can be addressed through medical or surgical intervention by a congenital heart team; however, comorbidities may complicate what is otherwise considered a “simple” diagnosis, transforming it into a high-risk perioperative challenge that requires multidisciplinary care. With more ACHD patients filling clinics and requiring operations, our case highlights the importance of high-complexity comprehensive centers developing protocols that deal with the comorbidities not frequently seen in the pediatric population which includes substance abuse.

## Supplementary Information

Supplementary Material 1.

## Data Availability

No datasets were generated or analysed during the current study.
